# Ultrasound-Guided Lumbar Plexus Injection: A Cadaveric Validation

**DOI:** 10.3390/diagnostics15233017

**Published:** 2025-11-27

**Authors:** Ondřej Naňka, Kamal Mezian, Ke-Vin Chang, Wei-Ting Wu, Vincenzo Ricci, Levent Özçakar

**Affiliations:** 1 Institute of Anatomy, First Faculty of Medicine, Charles University, 12800 Prague, Czech Republic; ondrej.nanka@lf1.cuni.cz; 2 Department of Rehabilitation Medicine, First Faculty of Medicine and General University Hospital in Prague, Charles University, 12800 Prague, Czech Republic; 3 Department of Physical Medicine and Rehabilitation, National Taiwan University Hospital, Bei-Hu Branch, Taipei 100225, Taiwan; 4 Department of Physical Medicine and Rehabilitation, National Taiwan University College of Medicine, Taipei 100229, Taiwan; 5 Center for Regional Anesthesia and Pain Medicine, Wang-Fang Hospital, Taipei Medical University, Taipei 116081, Taiwan; 6 Physical and Rehabilitation Medicine Unit, Luigi Sacco University Hospital, ASST Fatebenefratelli-Sacco, 20157 Milano, Italy; 7 Department of Physical and Rehabilitation Medicine, Hacettepe University Medical School, Ankara 06100, Turkey

**Keywords:** pain, sonography, lumbar plexus, spinal nerve, injection

## Abstract

Ultrasound (US) has gained increasing acceptance for evaluating the axial spine, including the lumbar region. While its accuracy for superficial structures such as facet joints and medial branches has been validated, evidence supporting its use for deeper targets, such as the lumbar plexus, remains limited. This cadaveric study aimed to assess the feasibility of US-guided lumbar plexus injection. A fresh-frozen female cadaver with a body mass index of 23 kg/m^2^, prepared using the “Fix-for-Life” technique, was utilized. Using a 2–5 MHz curved linear transducer (HS30; Samsung Medison, Seoul, Republic of Korea), injections were performed with an in-plane approach under continuous needle visualization. A 20-gauge, 7 cm spinal needle was used to deliver 5 mL of green dye targeting the L3 and L4 nerve roots. Dissection confirmed that L3 injection achieved dye spread to the extraforaminal region, whereas L4 injection demonstrated anterior dye distribution adjacent to the intervertebral foramen. The main limitations included the use of a single specimen and acoustic shadowing from articular processes, which impeded visualization of neural structures. This study demonstrates the feasibility of US-guided lumbar plexus injection and supports its potential application in clinical pain management, although further validation with larger sample sizes is warranted.

**Figure 1:**
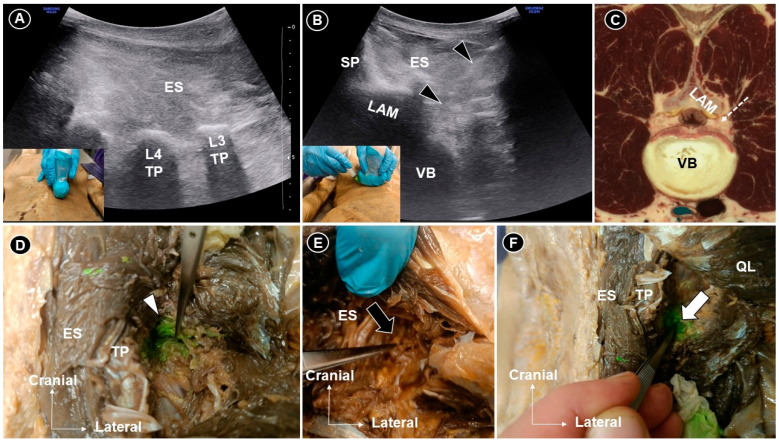
The transducer was placed sagittally to visualize the transverse process (**A**) and then rotated into the axial plane for an in-plane injection targeting the L3 nerve root (**B**). Corresponding cadaveric cross-sectional image (adapted with permission from the Visible Human Project^®^ and VH Dissector) (**C**). Dissection confirmed dye infiltration of the L3 rootlet (white arrowhead) at the extraforaminal level (**D**). For L4 injection, no dye was seen (black arrow) posterior or lateral to the foramen (**E**), whereas clear extension was observed (white arrow) adjacent to the intervertebral foramen (**F**). TP, transverse process; LAM, lamina; VB, vertebral body; ES, erector spinae; SP, spinous process; black arrowheads and white dashed arrow line indicate the needle trajectory. Ultrasound (US) has increasingly been applied in the evaluation of the axial spine, including the lumbar region. For superficial targets such as the facet joints and medial branches, its accuracy has been well validated [1,2]. However, studies systematically verifying its use for deeper structures, such as the lumbar plexus and intervertebral disks, remain limited. Given that lumbar radiculopathy affects 5–10% of patients with low back pain [3,4], lumbar plexus injection represents a valuable therapeutic option. This study aimed to assess the feasibility of US-guided lumbar plexus injection in a cadaveric model. A fresh-frozen female cadaver with a body mass index of 23 kg/m^2^, prepared using the “Fix-for-Life” technique, was utilized [5]. Each injection was performed using an in-plane approach, ensuring continuous visualization of the needle tip throughout the procedure. A 20-gauge, 7 cm spinal needle was used to deliver 5 mL of green dye under US guidance with a 2–5 MHz curved linear transducer (HS30; Samsung Medison Co., Ltd., Seoul, Republic of Korea). The probe was first positioned in the sagittal plane (**A**) at the midline and then sequentially moved to identify the spinous processes, laminae, facet joints, superior articular and transverse processes. The transverse processes served as landmarks for vertebral level identification. The right L3 and left L4 nerve roots were selected as targets. For L3 injection, the L3 and L4 transverse processes were initially identified. The transducer was then placed midway between them and rotated into the axial plane. In this view, the lamina and vertebral body were visualized. The needle was advanced in-plane from lateral to medial, toward the posterior edge of the L3 vertebral body (**B**,**C**), to the depth of the lamina. Injection was performed when the needle tip became obscured by the acoustic shadow of the lamina. For L4 injection, a similar technique was used, with the needle advanced approximately 1 cm further anteriorly, despite the loss of direct tip visualization. Cadaveric dissection confirmed that L3 injection resulted in dye infiltration of the L3 nerve rootlet at the extraforaminal level (**D**), with additional spread toward the foramen. For L4 injection, the dye was not evident around the root posterior or lateral to the foramen (**E**). However, after reflecting the quadratus lumborum and exposing the area anterior to the transverse process, the dye was clearly observed extending into the region adjacent to the intervertebral foramen (**F**). The variation in dye distribution likely resulted from procedural differences, particularly the greater needle advancement (approximately 1 cm) during the L4 root injection. This cadaveric study validated the feasibility of ultrasound-guided lumbar plexus injection, demonstrating both the anatomical plausibility and dye spread pattern under real-time ultrasound visualization. This innovative approach underscores the potential of US as a radiation-free alternative to fluoroscopy and computed tomography for targeting deep lumbar neural structures. Nonetheless, this study was limited by the use of a single cadaveric specimen, meaning that potential anatomic variability between specimens (e.g., lumbar sacralization or lumbosacralization) was not accounted for. Furthermore, acoustic shadowing from the articular processes limited the visualization of neural structures near the neural foramen under US imaging. This cadaveric validation demonstrates that US-guided lumbar plexus injection is feasible and can achieve dye spread to the lumbar nerve roots near the intervertebral foramen. While acoustic shadowing limits continuous needle visualization, careful landmark identification and trajectory adjustment enable effective targeting. We would like to emphasize that, during US-guided injection techniques, the posterior branches of the spinal nerve (dorsal rami) are more likely to be encountered than the anterior branches (ventral rami), given that the needle is advanced along a posterolateral-to-anteromedial trajectory. Nevertheless, when applying this technique in clinical practice, it is essential to aspirate before injection to ensure the absence of blood return, given the rich vascularity of the lumbar region and the proximity of segmental vessels coursing alongside the spinal nerves [6]. Activating power Doppler imaging at the target site may further assist in identifying and avoiding adjacent vascular structures. If corticosteroids are to be used, a non-particulate formulation is essential to preventing thromboembolic events in cases of accidental vascular puncture [7].

## Data Availability

The original contributions presented in the study are included in this article; further inquiries can be directed to the corresponding author.
